# Glucose Co-Treatment Modulates Caffeine-Induced Transcriptomic Alterations During Embryogenesis in Zebrafish (*Danio rerio*)

**DOI:** 10.3390/biology15161359

**Published:** 2026-08-10

**Authors:** Olivia M. Armstrong, Kara E. Stacy, Renfang S. Taylor

**Affiliations:** Anderson University, Anderson, IN 46001, USA; omarmstrong@anderson.edu (O.M.A.); kestacy@anderson.edu (K.E.S.)

**Keywords:** caffeine, glucose, RNA-seq, transcriptomic, development, zebrafish embryo

## Abstract

Caffeine exposure during pregnancy has been associated with developmental abnormalities; however, the combined effects of caffeine and glucose on embryogenesis remain unclear. In this study, zebrafish embryos were exposed to caffeine or caffeine plus glucose co-treatment from 3 h post-fertilization to 96 h post-fertilization to evaluate developmental, physiological, and transcriptomic changes. Morphological analyses revealed developmental abnormalities in treatment groups, including pericardial edema, abnormal body curvature, delayed tail extension, and enlarged yolk sac morphology. Significant alterations in hatch rate and heart rate were also observed following treatment exposure. RNA sequencing analysis demonstrated that caffeine induced broad transcriptomic disruption involving neuronal signaling, embryonic development, metabolism, mitochondrial activity, immune regulation, and protein turnover pathways. Compared with caffeine treatment alone, caffeine plus glucose co-treatment was associated with differences in differential gene expression and developmental and metabolic signaling pathways. Alternative splicing analysis further identified widespread exon-skipping events and altered splicing patterns in genes associated with developmental and neuronal regulation. Collectively, these findings indicate that glucose co-treatment was associated with altered developmental and transcriptomic responses relative to caffeine exposure alone during zebrafish embryogenesis. Because a glucose-only treatment was not included in the present study, these findings should not be interpreted as demonstrating a definitive caffeine–glucose interaction.

## Simple Summary

Caffeine is one of the most widely consumed stimulants worldwide and is commonly found in coffee, tea, soft drinks, and energy drinks. Excessive caffeine intake during pregnancy has been associated with adverse developmental outcomes, but little is known about how glucose influences caffeine-associated molecular responses during embryonic development. In this study, zebrafish embryos, a well-established vertebrate model for developmental research, were exposed to caffeine or caffeine combined with glucose. Caffeine exposure caused developmental abnormalities, including swelling around the heart, abnormal body shape, delayed growth, and changes in heart rate and hatching. RNA sequencing revealed that caffeine altered genes and biological pathways involved in nervous system development, metabolism, energy production, cellular growth, and RNA splicing. Compared with caffeine treatment alone, embryos exposed to caffeine plus glucose exhibited distinct transcriptomic and alternative splicing profiles, indicating differences in molecular responses during development. Because a glucose-only treatment group was not included in the present study, these findings should not be interpreted as demonstrating a definitive interaction between caffeine and glucose. Instead, they provide new insight into the molecular changes associated with caffeine exposure and identify transcriptomic differences associated with glucose co-treatment during early vertebrate development.

## 1. Introduction

Caffeine is one of the most widely consumed psychoactive substances worldwide and is commonly present in beverages such as coffee, tea, soft drinks, energy drinks, and chocolate-containing products [1]. Moderate caffeine consumption has been associated with beneficial physiological effects, including increased alertness, enhanced cognitive performance, antioxidant activity, and reduced risk of certain cardiometabolic diseases [2,3,4]. However, excessive caffeine intake has raised concerns regarding adverse health outcomes, particularly during pregnancy and early embryonic development [5,6,7,8,9,10,11]. Recent reviews have further emphasized that physiological changes during pregnancy alter caffeine metabolism, resulting in prolonged caffeine half-life and increased fetal exposure throughout gestation [12]. In addition, maternal caffeine consumption has been associated with an increased risk of adverse birth outcomes, including delivery of small-for-gestational-age infants and altered fetal growth trajectories [13].

During pregnancy, caffeine readily crosses the placental barrier and can accumulate in fetal tissues because the developing fetus has limited capacity to metabolize caffeine [5,6]. Epidemiological studies and meta-analyses have linked excessive maternal caffeine consumption to adverse pregnancy outcomes, including miscarriage, fetal growth restriction, low birth weight, preterm birth, gestational hypertension, and preeclampsia [7,8,9]. An integrative review by Silva et al. further summarized evidence supporting associations between maternal caffeine intake and adverse reproductive and developmental outcomes, while highlighting the need for continued investigation of underlying biological mechanisms [14]. Recent epigenetic studies further suggest that prenatal caffeine exposure may induce long-term alterations in Deoxyribonucleic acid (DNA) methylation and developmental programming, potentially affecting offspring health beyond embryonic development [10]. Experimental evidence indicates that caffeine may influence embryogenesis through alterations in oxidative stress, cardiovascular physiology, cellular metabolism, angiogenesis, and gene regulation [5,11].

The zebrafish (*Danio rerio*) is a well-established vertebrate model for investigating embryonic development, toxicology, genetics, and human disease because of its rapid external development, optical transparency, high fecundity, and extensive genetic conservation with humans [15,16]. Previous studies have demonstrated that caffeine exposure can induce developmental abnormalities in zebrafish embryos, including altered locomotor activity, impaired neuromuscular development, angiogenic defects, cardiac dysfunction, behavioral changes, and oxidative stress responses [17,18,19,20,21,22,23]. Caffeine has also been shown to alter cardiac gene expression, skeletal development, and developmental signaling pathways during embryogenesis [23,24]. These findings suggest that caffeine may influence multiple biological processes during early vertebrate development.

In addition to caffeine exposure, maternal metabolic disorders such as gestational diabetes mellitus (GDM) represent major risk factors for adverse fetal development [25]. Hyperglycemia during pregnancy has been associated with congenital abnormalities, altered organogenesis, cardiovascular defects, and long-term metabolic dysfunction in offspring [25]. To model these conditions experimentally, zebrafish embryos have been widely utilized to investigate the effects of glucose dysregulation on embryonic development and metabolism [26].

Our laboratory previously demonstrated that high glucose exposure disrupts zebrafish embryogenesis by delaying hatching, altering heart rate, modifying embryonic morphology, and dysregulating multiple developmental pathways, including oxidative phosphorylation, glycolysis/gluconeogenesis, Wnt signaling, and Notch signaling [27]. More recently, we reported that high-salt exposure induced cardiovascular developmental abnormalities through disruption of calcium signaling and Mitogen-Activated Protein Kinase (MAPK) pathways during embryogenesis [28]. Together, these studies suggest that environmental and metabolic stressors converge on common developmental signaling networks that regulate cardiovascular function, energy metabolism, and embryonic growth.

Although the developmental effects of caffeine and glucose have been studied independently, relatively little is known about how glucose influences caffeine-induced transcriptomic responses during embryogenesis. Given the widespread consumption of caffeine and the increasing prevalence of gestational diabetes worldwide, understanding the interaction between caffeine exposure and altered glucose metabolism is of considerable biological and public health significance.

Therefore, the objective of the present study was to investigate the effects of caffeine and caffeine-plus-glucose co-treatment on zebrafish embryonic development using phenotypic assessments and transcriptomic analyses. Hatch rate, heart rate, morphology, differential gene expression, pathway enrichment, and alternative splicing events were examined to determine how glucose co-treatment modifies caffeine-induced developmental responses. We hypothesized that glucose co-treatment would significantly alter caffeine-induced transcriptomic signatures and developmental pathways associated with metabolism, cardiovascular development, and embryonic growth.

## 2. Materials and Methods

### 2.1. Zebrafish Husbandry and Embryo Collection

Adult fish were housed in a recirculating aquatic system at 28.5 °C under a 14 h light/10 h dark photoperiod. Fertilized embryos were obtained through natural spawning and collected within 3 h post-fertilization (hpf). Embryos were examined microscopically, and only normally fertilized embryos were selected for subsequent experiments.

### 2.2. Experimental Design and Treatments

Building upon our previous investigations of glucose-induced developmental dysregulation and salt-induced cardiovascular abnormalities in zebrafish embryos [27,28], embryos were randomly assigned to one of three treatment groups: 1. Control group: embryo medium only (Embryo Rearing Solution, Laboratory Grade, Cat. No. 859450; Carolina Biological Supply Company, Burlington, NC, USA); 2. Caffeine group (0.15 mg/mL caffeine: ReagentPlus^®^, Cat. No. C0750, Sigma-Aldrich, St. Louis, MO, USA); 3. Caffeine plus glucose group (0.15 mg/mL caffeine + 3% glucose: D-(+)-Glucose, Cat. No. G7021; CAS No. 50-99-7; purity ≥ 99.5%; Sigma-Aldrich, St. Louis, MO, USA). The caffeine concentration (0.15 mg/mL) was selected based on previous zebrafish studies demonstrating developmental and physiological effects without excessive embryonic mortality [21,22]. The 3% glucose concentration was selected based on our previous study using the same zebrafish embryo model, which demonstrated significant developmental and transcriptomic responses [27].

Treatment solutions were prepared fresh and renewed every 24 h throughout the experimental period. Embryos were exposed continuously from 3 hpf until 96 hpf. Each treatment consisted of three Petri dishes (biological replicates), each containing 29 embryos. All embryos were obtained from a single spawning event (one clutch) and were randomly assigned to treatment groups after selection of normally fertilized embryos. Unfertilized embryos and embryos exhibiting obvious developmental abnormalities before treatment initiation were excluded. Embryonic development was monitored daily throughout the exposure period.

### 2.3. Morphological Assessment, Hatch Rate, and Heart Rate Measurements

Embryos were examined at 24, 48, 72, and 96 hpf using a stereomicroscope. Morphological observations included body shape, developmental progression, edema formation, and overall appearance. Representative images were captured for each treatment group.

Morphological observations, survival, and hatch rate were recorded for all embryos in each biological replicate. Heart rate was measured using three randomly selected embryos from each Petri dish at 72 and 96 hpf by counting cardiac contractions under microscopic observation. The average heart rate from the three embryos in each replicate was used for statistical analysis.

### 2.4. RNA Extraction and Sequencing

At 96 hpf, embryos from each treatment group were collected for transcriptomic analysis. Total RNA was extracted using the Quick-RNA Miniprep Plus Kit (Cat. No. R1057T; Zymo Research, Irvine, CA, USA) according to the manufacturer’s instructions and our previously published protocol [27,28]. RNA libraries were prepared and sequenced by Novogene Corporation (Sacramento, CA, USA). Total RNA was extracted from all surviving embryos collected from each biological replicate at the designated sampling time point. RNA quality and integrity were evaluated using an Agilent 2100 system (Agilent Technologies, Santa Clara, CA, USA) prior to library construction. All RNA samples passed Novogene quality control, with RNA integrity numbers (RINs) ranging from 7.7 to 9.5. RNA-seq libraries were constructed using the VAHTS Universal V10 RNA-seq Library Prep Kit for Illumina (Cat. No. NR605; Vazyme Biotech Co., Ltd., Nanjing, China) and sequenced on an Illumina NovaSeq X Plus platform (Illumina, San Diego, CA, USA) using 150-bp paired-end sequencing. Approximately 9 Gb of raw sequencing data were generated per sample, yielding an average of approximately 76 million clean reads (11.3 Gb clean bases) per sample. Sequencing quality was high, with Q20 values greater than 99% and Q30 values greater than 96% for all samples.

### 2.5. Differential Gene Expression Analysis

Clean reads were aligned to the Danio rerio reference genome (GRCz11) using HISAT2, and transcript abundance was quantified. Differential gene expression analysis was performed using the DESeq2 package (v1.42.0) based on the negative binomial distribution model. Gene expression values were normalized using the DESeq2 normalization method. Three pairwise comparisons were performed: Caffeine vs. Control, Caffeine + Glucose vs. Control, and Caffeine + Glucose vs. Caffeine. Volcano plots, Venn diagrams, and hierarchical clustering analyses were generated to visualize transcriptomic differences among treatment groups.

### 2.6. Gene Ontology and KEGG Pathway Enrichment Analysis

Gene Ontology (GO) enrichment and Kyoto Encyclopedia of Genes and Genomes (KEGG) pathway enrichment analyses were performed using the standardized bioinformatics pipeline provided by Novogene. Differentially expressed genes identified in each pairwise comparison were analyzed against the annotated Danio rerio reference genome (GRCz11) as the background gene set. Complete GO and KEGG enrichment result tables are available in the NCBI Gene Expression Omnibus (GEO) under accession number GSE334680.

### 2.7. Alternative Splicing Analysis

Alternative splicing events were analyzed from RNA sequencing data to identify transcriptomic changes associated with caffeine exposure and glucose co-treatment. Splicing categories examined included skipped exons (SE), alternative 5′ splice sites (A5SS), alternative 3′ splice sites (A3SS), mutually exclusive exons (MXE), and retained introns (RI). Only reads spanning splice junctions were used to evaluate differential splicing events. Developmentally relevant genes exhibiting differential splicing patterns were further evaluated and visualized using heatmaps and sashimi plots.

### 2.8. Statistical Analysis

Data are presented as mean ± standard deviation (SD). Statistical analyses of developmental phenotypes were performed using GraphPad Prism version 10.1.2 (GraphPad Software, San Diego, CA, USA). Differences among treatment groups were analyzed using one-way analysis of variance (ANOVA) followed by Tukey’s multiple-comparison test. A *p* value < 0.05 was considered statistically significant.

For RNA-sequencing analysis, gene expression levels were quantified and differential expression analysis was performed using DESeq2. Differentially expressed genes (DEGs) were defined as those with an adjusted *p* value (false discovery rate, FDR) ≤ 0.05 and an absolute log_2_ fold change > 1. *p* values were adjusted for multiple testing using the Benjamini–Hochberg (BH) false discovery rate (FDR) procedure.

Gene Ontology (GO) enrichment and Kyoto Encyclopedia of Genes and Genomes (KEGG) pathway analyses were considered statistically significant at an adjusted *p* value (FDR) ≤ 0.05 following Benjamini–Hochberg correction. Differential alternative splicing events were identified using the rMATS statistical framework with an FDR < 0.05.

## 3. Results

### 3.1. Developmental Effects of Caffeine and Glucose Co-Treatment

#### 3.1.1. Morphology Changes

**Figure 1:**
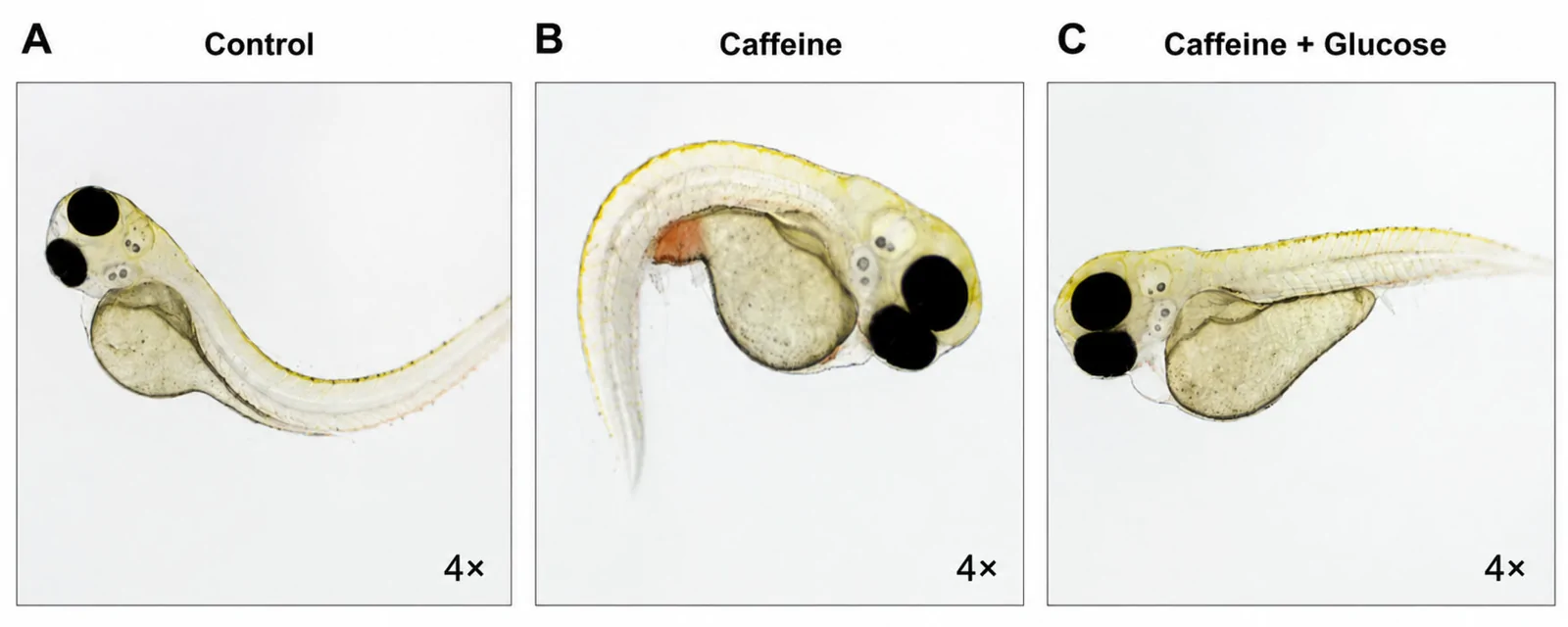
Representative images of zebrafish embryos at 96 hpf. (**A**) Control group. (**B**) Caffeine-treated group. (**C**) Caffeine plus glucose co-treatment group. Images were captured using a stereomicroscope at 4× magnification.

Morphological examination revealed several developmental abnormalities in caffeine-treated and caffeine plus glucose-treated embryos compared to controls (Figure 1). Embryonic developmental stages were evaluated according to established zebrafish staging criteria [15]. Control embryos exhibited normal developmental progression, whereas treated embryos displayed delayed development, enlarged yolk sacs, body curvature, and pericardial edema. These abnormalities were more apparent in the caffeine-treated group and remained evident in the caffeine plus glucose co-treatment group.

#### 3.1.2. Hatch Rate and Heart Rate

**Figure 2:**
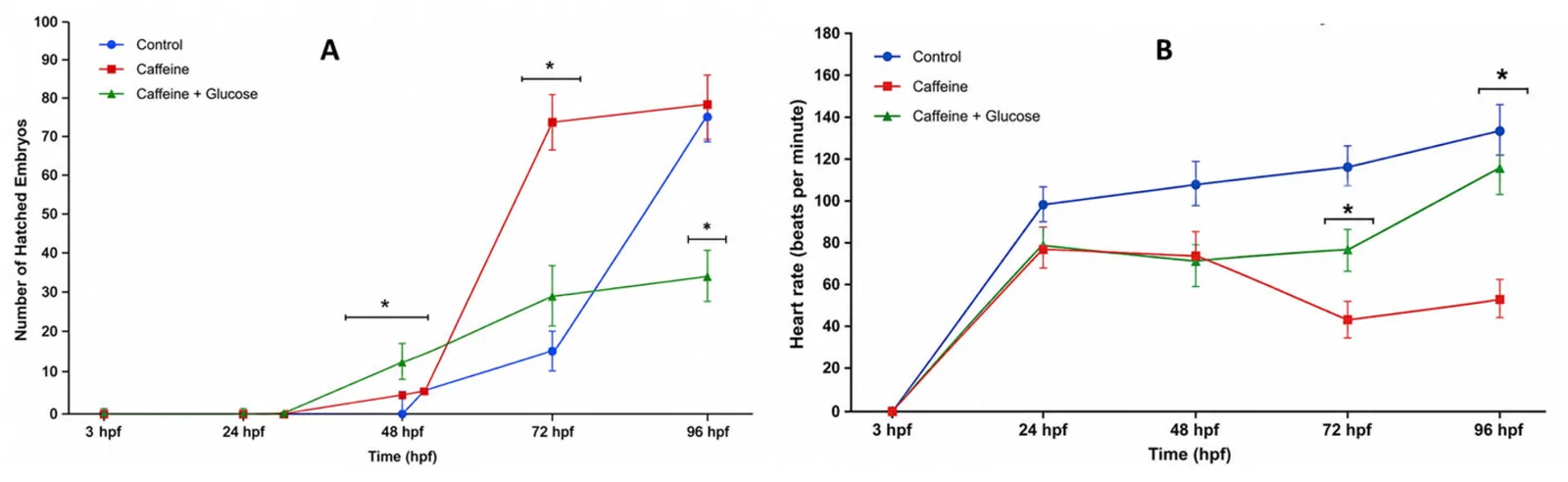
Effects of caffeine and glucose treatments on zebrafish embryonic development. (**A**) Hatch rate of zebrafish embryos at different developmental time points. Hatch rate was determined using all embryos from three biological replicates (29 embryos per replicate). (**B**) Heart rate of zebrafish embryos at 72 and 96 hpf. Asterisks (*) indicate statistically significant differences compared with the control group (*p* < 0.05).

Caffeine exposure significantly affected hatch rate and heart rate during embryonic development (Figure 2). Heart rate and developmental progression are commonly used indicators of embryonic health and developmental toxicity in zebrafish models [15,21]. Both caffeine-treated and caffeine plus glucose-treated embryos exhibited delayed hatching and altered cardiac function compared to controls. The caffeine plus glucose group demonstrated partial modification of caffeine-induced physiological responses but did not completely restore normal developmental parameters.

### 3.2. Transcriptomic Responses to Caffeine and Glucose Co-Treatment

#### 3.2.1. RNA Sequencing Quality Assessment

**Figure 3:**
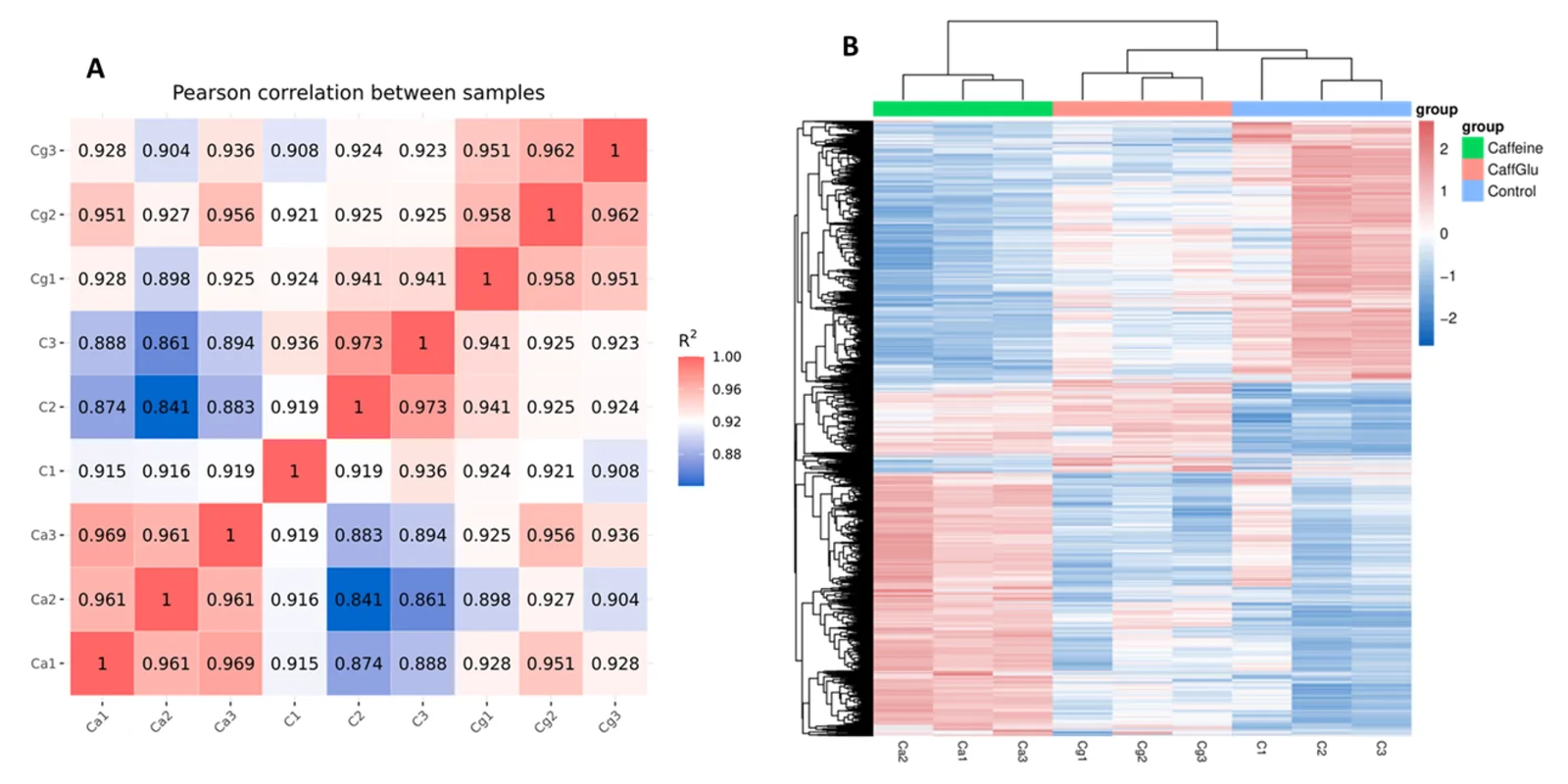
Pearson correlation and hierarchical clustering heatmap of RNA-seq samples. (**A**) Pearson correlation analysis among samples. CA represents the caffeine-treated group, C represents the control group, and CG represents the caffeine plus glucose co-treatment group. (**B**) Hierarchical clustering heatmap showing the biological triplicates among the three experimental groups. CaffGlu represents the caffeine plus glucose co-treatment group.

Pearson correlation analysis and hierarchical clustering demonstrated strong reproducibility among biological replicates and clear separation among experimental groups (Figure 3). Replicates within each treatment group clustered closely together, indicating high consistency in gene expression profiles. Distinct clustering patterns were observed among the control, caffeine-treated, and caffeine plus glucose-treated groups, demonstrating that both caffeine exposure and glucose co-treatment induced substantial transcriptomic alterations during zebrafish embryogenesis.

#### 3.2.2. Differential Gene Expression Analysis

**Figure 4:**
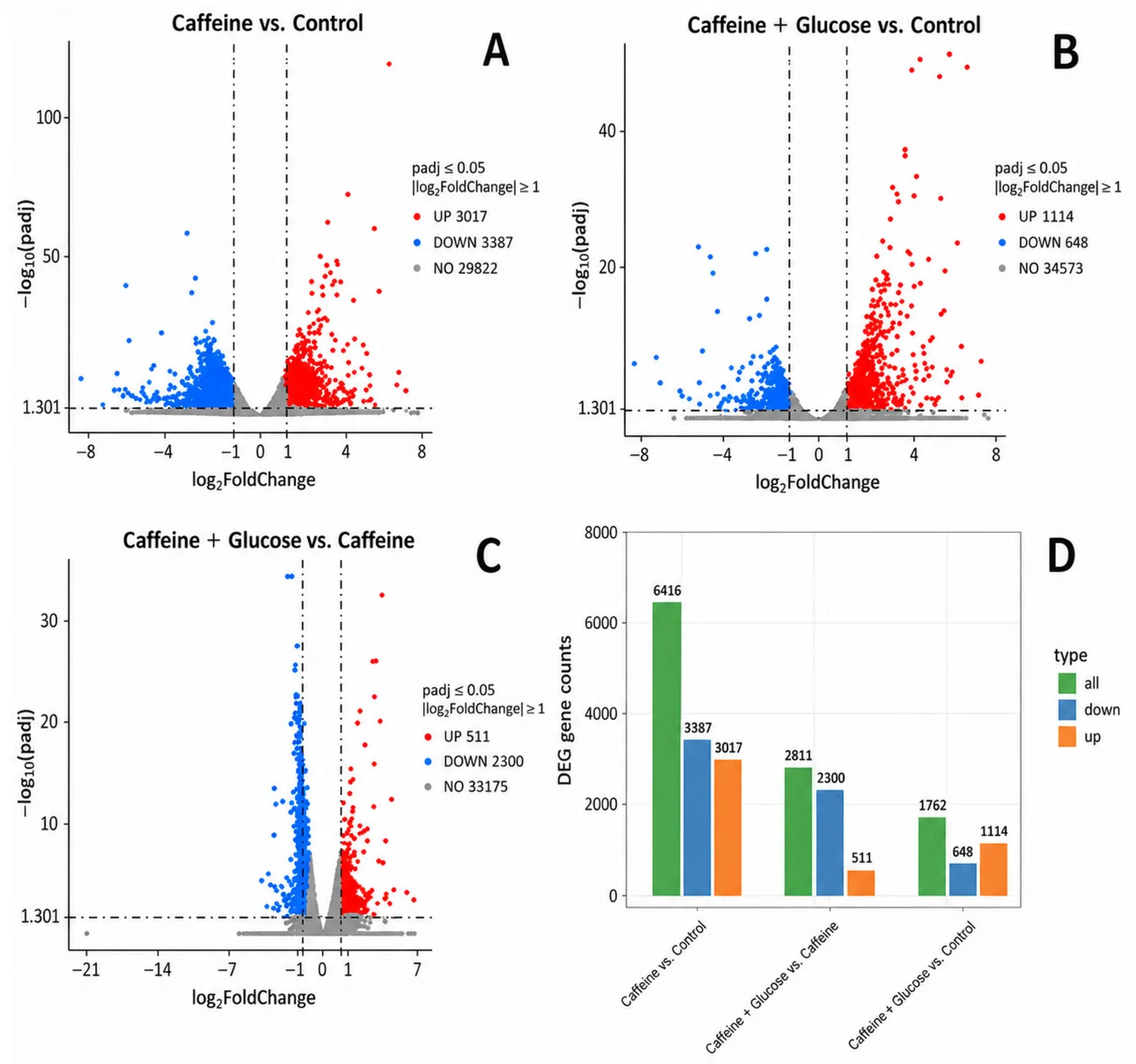
Volcano plots showing significantly upregulated and downregulated genes identified by RNA-seq analysis. (**A**) Caffeine-treated group versus control group. (**B**) Caffeine plus glucose co-treatment group versus control group. (**C**) Caffeine plus glucose co-treatment group versus caffeine-treated group. Red dots represent significantly upregulated genes, while blue dots represent significantly downregulated genes. (**D**) Number of significantly differentially expressed genes identified in the three comparison groups.

Volcano plot analysis identified substantial transcriptomic changes among treatment groups (Figure 4). Caffeine exposure alone induced extensive differential gene expression compared with the control group, with 3017 upregulated genes and 3387 downregulated genes identified. Similarly, the caffeine plus glucose co-treatment group exhibited 1114 upregulated genes and 648 downregulated genes relative to the control group. When the caffeine plus glucose group was compared directly with the caffeine-treated group, 511 genes were upregulated and 2300 genes were downregulated. Overall, caffeine exposure alone produced the greatest transcriptomic disruption, whereas glucose co-treatment reduced the magnitude of differential gene expression relative to caffeine exposure alone (Figure 4).

**Figure 5:**
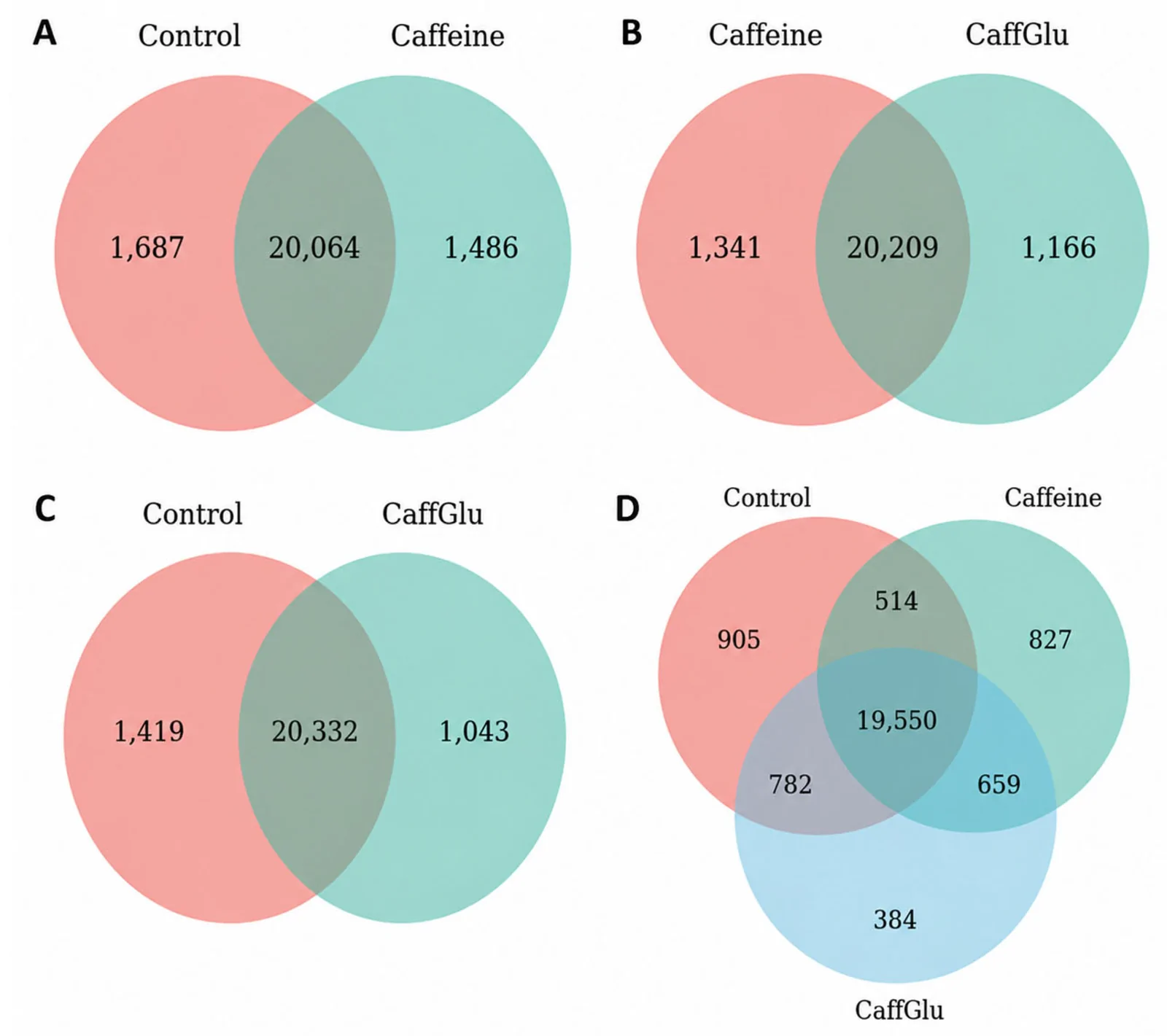
Venn diagrams showing the overlap and unique expressed genes detected among the three treatment groups. (**A**) Control versus caffeine-treated group. (**B**) Caffeine-treated group versus caffeine plus glucose co-treatment group. (**C**) Control versus caffeine plus glucose co-treatment group. (**D**) Overlap of expressed genes detected among the control, caffeine-treated, and caffeine plus glucose co-treatment groups. The diagrams illustrate the large proportion of genes commonly expressed among treatment groups, together with genes uniquely detected in each group, reflecting overall transcriptome composition rather than differential gene expression.

Venn diagram analysis demonstrated substantial overlap in expressed genes among the treatment groups (Figure 5). A total of 20,064 genes were commonly detected between the control and caffeine-treated groups, while 20,332 genes were shared between the control and caffeine plus glucose co-treatment groups. Similarly, 20,209 genes were shared between the caffeine-treated and caffeine plus glucose co-treatment groups. Across all three treatment groups, 19,550 genes were commonly expressed, indicating that the majority of the zebrafish embryonic transcriptome was shared despite treatment exposure. In addition, each treatment group contained a smaller set of uniquely detected genes, suggesting treatment-associated differences in overall gene expression profiles. Figure 5 illustrates the overlap of expressed genes and should not be interpreted as representing differentially expressed genes identified by DESeq2 analysis.

#### 3.2.3. Top Differentially Expressed Genes

Analysis of the top 10 upregulated and top 10 downregulated genes revealed distinct functional categories associated with neuronal signaling, embryonic development, metabolism, immune regulation, protein turnover, and chromatin regulation (Table 1). Among the top differentially expressed genes in the caffeine versus control comparison were cnih2, lepb, irf4b, il12rb2, and igf1ra, whereas he1a and he1b were among the most strongly downregulated genes. Comparison of the caffeine plus glucose co-treatment group with the caffeine-treated group identified additional highly regulated genes involved in developmental regulation, metabolism, and chromatin organization.

#### 3.2.4. Gene Ontology Enrichment Analysis

**Figure 6:**
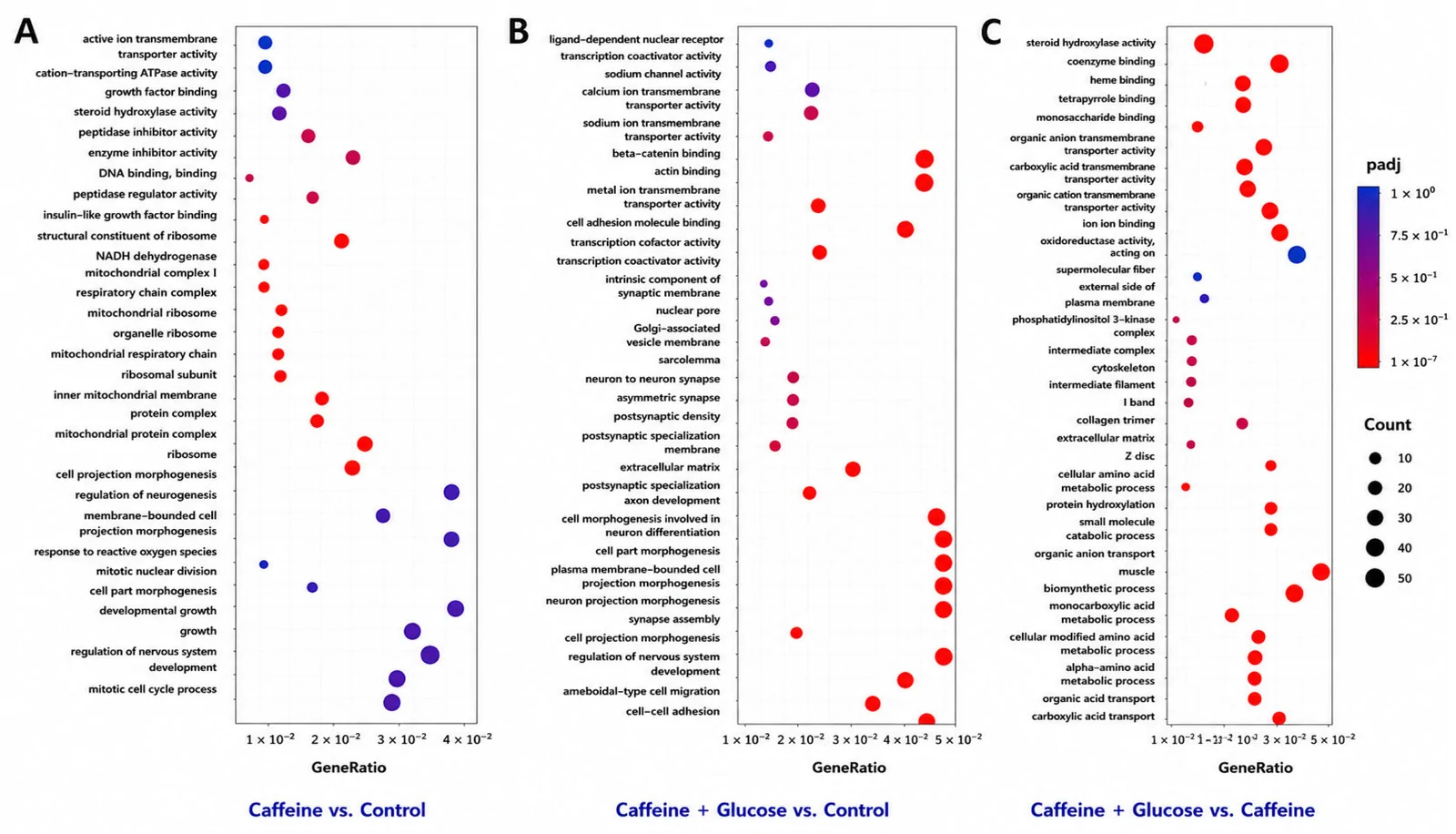
Gene Ontology (GO) enrichment analysis of differentially expressed genes (DEGs). (**A**) Caffeine vs. Control. (**B**) Caffeine + Glucose vs. Control. (**C**) Caffeine + Glucose vs. Caffeine. The x-axis represents GeneRatio, bubble size indicates the number of DEGs (Count), bubble color represents the adjusted *p* value (padj), and the y-axis lists the enriched GO terms.

GO enrichment analysis revealed significant alterations in biological process, cellular component, and molecular function categories across all comparisons (Figure 6). Enriched GO terms in the caffeine versus control comparison were primarily associated with ribosome biogenesis, translation, mitochondrial activity, and developmental processes. Glucose co-treatment altered enrichment patterns related to metabolism, extracellular matrix organization, cell adhesion, and developmental regulation.

#### 3.2.5. KEGG Pathway Enrichment Analysis

**Figure 7:**
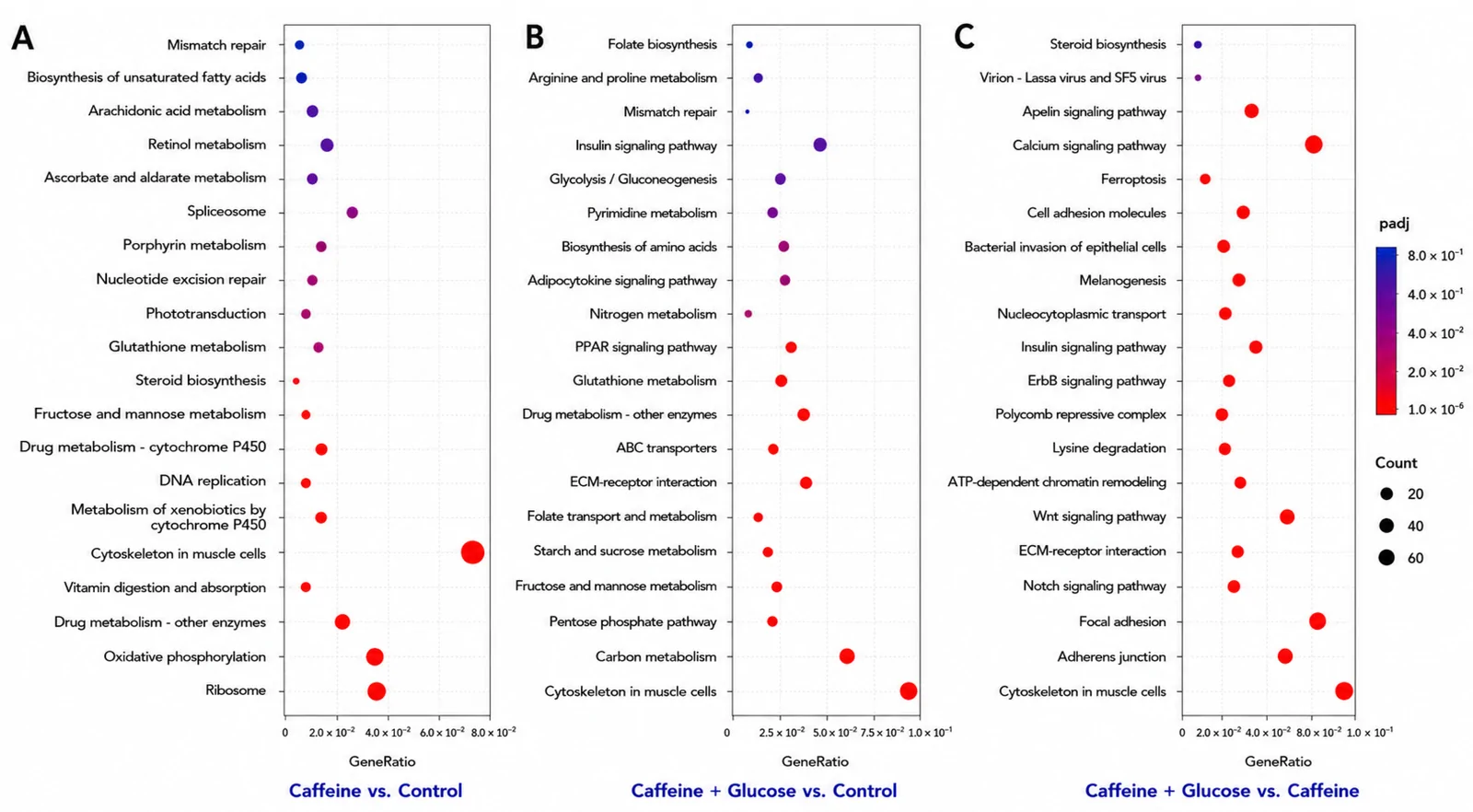
KEGG pathway enrichment analysis of differentially expressed genes (DEGs). (**A**) Caffeine vs. Control. (**B**) Caffeine + Glucose vs. Control. (**C**) Caffeine + Glucose vs. Caffeine. The x-axis represents GeneRatio. Bubble size indicates the number of DEGs (Count), and bubble color represents the adjusted *p* value (padj).

KEGG pathway analysis demonstrated substantial pathway alterations associated with caffeine exposure and glucose co-treatment (Figure 7). In the caffeine versus control comparison, the most significantly enriched pathways included ribosome, oxidative phosphorylation, drug metabolism, vitamin digestion and absorption, and cytoskeleton in muscle cells. The caffeine plus glucose versus control comparison showed enrichment of pathways related to ribosome, oxidative phosphorylation, proteasome, lysosome, and spliceosome function. Direct comparison of the caffeine plus glucose and caffeine-treated groups identified enrichment of pathways associated with focal adhesion, extracellular matrix–receptor interaction, adherens junctions, and developmental signaling pathways.

#### 3.2.6. Alternative Splicing Analysis

**Figure 8:**
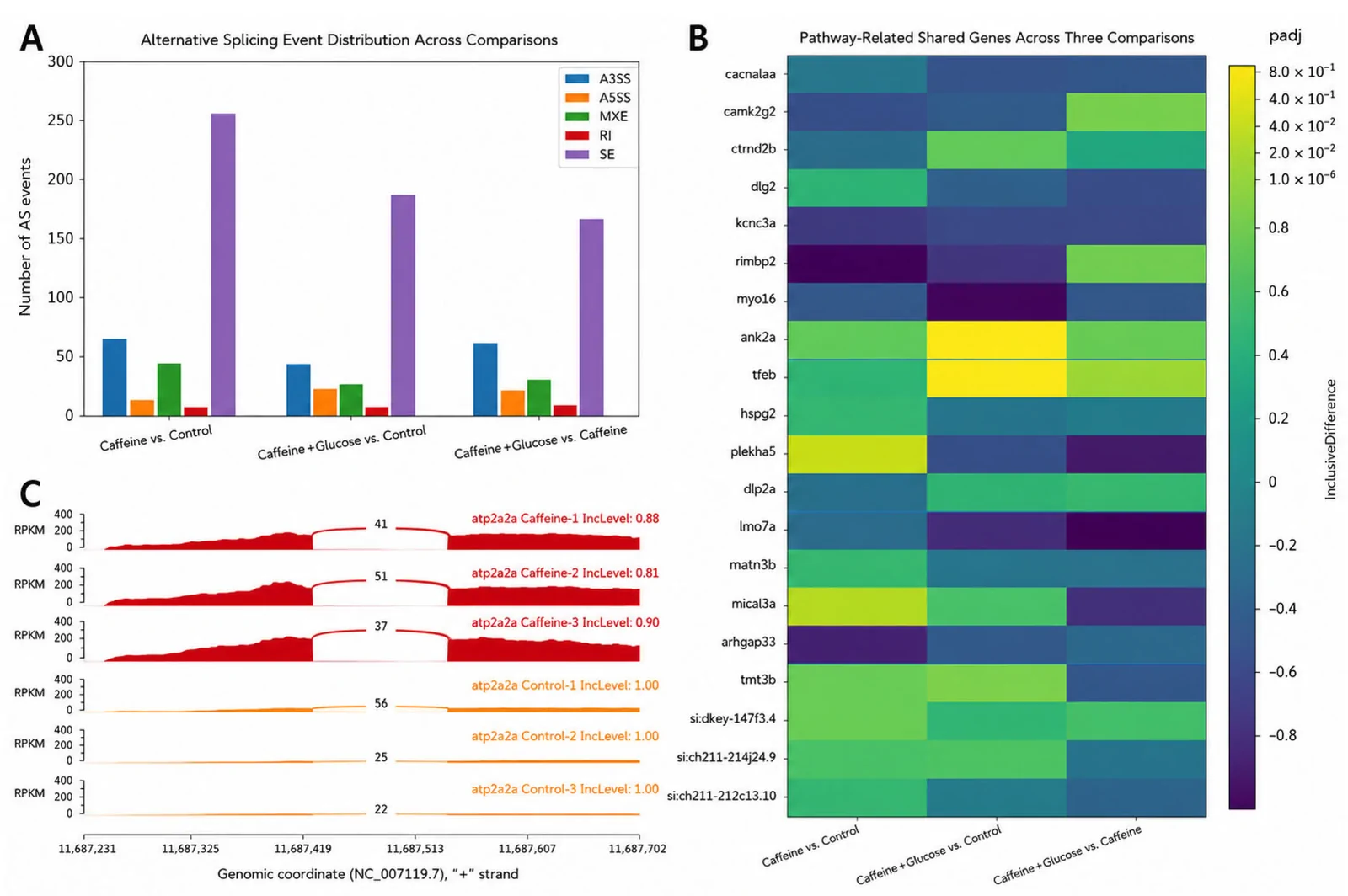
Alternative splicing (AS) analysis of RNA-seq data among the treatment groups. (**A**) Distribution of alternative splicing events, including alternative 3′ splice site (A3SS), alternative 5′ splice site (A5SS), mutually exclusive exons (MXE), retained introns (RI), and skipped exons (SE), identified in the three comparison groups. (**B**) Heatmap showing the expression patterns of 20 genes associated with developmental pathways among the three comparison groups. (**C**) Visualization of the ATP2a2a gene exhibiting an A3SS event in the caffeine plus glucose co-treatment group versus the control group, where red represents the treatment group and orange represents the control group.

Alternative splicing analysis revealed extensive transcriptomic remodeling following caffeine exposure and glucose co-treatment (Figure 8). Skipped exon (SE) events represented the predominant splicing category across all comparisons, followed by alternative 3′ splice site (A3SS), alternative 5′ splice site (A5SS), mutually exclusive exon (MXE), and retained intron (RI) events. Several genes involved in calcium signaling, neuronal function, embryonic development, and cellular regulation exhibited differential splicing patterns among treatment groups. Representative alternatively spliced genes included atp2a2a, cacna1aa, tfeb, and polg2, indicating widespread effects of caffeine and glucose exposure on post-transcriptional regulation during zebrafish embryogenesis.

## 4. Discussion

The present study demonstrated that caffeine exposure induced significant developmental, physiological, and transcriptomic alterations during zebrafish embryogenesis, while glucose co-treatment partially modified these responses. Morphological abnormalities observed in the treatment groups, including pericardial edema, abnormal body curvature, delayed tail extension, and enlarged yolk sac morphology, suggest that caffeine exposure associated with disrupted embryonic and cardiovascular development. Similar developmental defects have previously been reported in zebrafish embryos following caffeine exposure, including impaired angiogenesis, developmental delay, cardiovascular abnormalities, and movement disorders [17,18,19,20]. Rana et al. demonstrated that caffeine exposure significantly alters zebrafish embryonic heart rate through calcium-associated signaling pathways [21], while Abdelkader et al. reported altered heartbeat and cell damage-related gene expression during early developmental stages [22]. Fang et al. further showed that caffeine exposure modifies cardiac developmental gene expression in embryonic cardiomyocytes [23]. These findings are consistent with the altered hatch rate, heart rate, and cardiovascular abnormalities observed in the present study.

RNA-seq analysis revealed that caffeine exposure alone induced the greatest transcriptomic disruption among the treatment groups, suggesting broad molecular dysregulation during embryonic development. Differentially expressed genes were strongly associated with neuronal signaling, embryogenesis, metabolism, mitochondrial function, immune regulation, and protein turnover pathways. Particularly notable was the suppression of hatching-associated genes he1a and he1b together with altered expression of neuronal signaling genes such as cnih2 and nr4a3. Previous studies have demonstrated that caffeine exposure induces neuromuscular and developmental abnormalities in zebrafish embryos [17], while Stengel et al. emphasized the importance of neuronal signaling pathways during zebrafish neurotoxicity assessment [29]. White et al. identified dynamic transcriptomic regulation during zebrafish embryogenesis [30], supporting the sensitivity of developmental signaling pathways during early vertebrate development. Amsterdam et al. similarly identified numerous genes essential for vertebrate embryonic development and organogenesis [31]. The altered neuronal-associated genes identified in the current study therefore suggest that caffeine exposure may interfere with neural signaling and developmental communication pathways during embryogenesis.

GO and KEGG enrichment analyses demonstrated that caffeine exposure strongly affected oxidative phosphorylation, ribosomal activity, spliceosome regulation, and metabolic pathways. Mitochondrial activity and oxidative phosphorylation are essential for embryonic energy production and organogenesis, particularly during periods of rapid cellular growth and differentiation. Disruption of these pathways may be associated with the developmental delay and physiological abnormalities observed in the treatment groups. Dhillon et al. characterized dynamic metabolic changes throughout zebrafish embryogenesis [32], while Konadu et al. demonstrated that altered glucose exposure significantly affects metabolic and appetite-related gene expression during zebrafish development [26]. Our previous study demonstrated that high glucose exposure disrupts zebrafish embryological development through modulation of Wnt signaling and metabolic pathways [27]. In addition, we recently reported that high-salt exposure induces cardiovascular developmental abnormalities through disruption of calcium signaling and MAPK pathways [28]. Together, these findings suggest that diverse environmental and metabolic stressors may affect common developmental signaling networks that regulate embryonic growth, cardiovascular development, and energy metabolism. The enrichment of proteasome and lysosomal pathways is consistent with altered cellular stress responses and protein degradation processes following caffeine exposure.

An important finding of the present study was that zebrafish embryos exposed to caffeine plus glucose exhibited transcriptomic profiles that differed from those exposed to caffeine alone. Compared with caffeine exposure alone, the caffeine plus glucose group exhibited fewer differentially expressed genes and distinct pathway enrichment associated with extracellular matrix organization, metabolism, developmental signaling, and mitochondrial regulation. These findings suggest that glucose co-treatment was associated with altered molecular responses during caffeine exposure. However, because a glucose-only treatment group was not included in the present study, it is not possible to distinguish glucose-specific effects from true caffeine–glucose interaction effects within the same experimental framework. Previous studies have shown that high-glucose environments independently alter embryonic signaling pathways, oxidative stress responses, and developmental progression in zebrafish embryos [26,27]. Therefore, the transcriptomic differences observed between the caffeine and caffeine plus glucose groups should be interpreted with caution and may reflect glucose-specific effects, altered physiological responses during co-treatment, or potential interaction effects that require further investigation. Because many caffeinated beverages consumed worldwide also contain high levels of sugar, these findings provide insight into transcriptomic differences associated with combined caffeine and glucose exposure during embryogenesis. Future studies incorporating a glucose-only treatment group within the same experimental design will be necessary to determine the specific contribution of glucose and to establish whether true interaction effects exist.

Alternative splicing analysis demonstrated widespread transcriptomic remodeling following caffeine exposure and glucose co-treatment. Skipped exon events represented the predominant alternative splicing category across all treatment comparisons, while genes associated with calcium regulation, developmental signaling, and neuronal pathways displayed altered splicing patterns. The ATP2a2a gene, which plays an important role in calcium transport and cardiac muscle function, exhibited altered exon inclusion patterns in treatment groups. Alternative splicing is a critical regulatory mechanism during vertebrate embryogenesis and contributes to tissue differentiation, developmental timing, and organogenesis [16,30]. Increasing evidence suggests that environmental stressors and metabolic disturbances can alter RNA processing and spliceosomal regulation during development. Therefore, altered splicing patterns may be associated with the developmental and physiological abnormalities observed following caffeine exposure.

Caffeine exerts diverse physiological effects in both humans and vertebrate animal models through modulation of adenosine receptor signaling, neurotransmitter activity, cardiovascular function, metabolism, and oxidative stress responses. Previous reviews have highlighted both beneficial and adverse effects of caffeine depending on dose, developmental stage, and duration of exposure, emphasizing the importance of understanding caffeine-mediated physiological regulation during development [33,34,35]. In addition, long-term exposure studies in zebrafish have demonstrated that environmentally relevant caffeine concentrations can alter behavioral responses, biochemical parameters, and physiological homeostasis, suggesting that caffeine may exert persistent effects on multiple biological systems [36]. These findings support the broad transcriptomic alterations observed in the present study and further indicate that caffeine-induced developmental effects may involve coordinated changes across metabolic, neurological, and cardiovascular pathways.

The findings of this study also support growing epidemiological and experimental evidence suggesting that prenatal caffeine exposure may influence fetal growth, cardiovascular development, metabolism, and neurodevelopment. Previous studies have associated maternal caffeine intake with altered implantation, placentation, fetal growth, low birth weight, childhood obesity, gestational hypertension, preeclampsia, preterm birth, and other adverse developmental outcomes [1,2,4,5,6,7,8,9,10]. Together, these studies support the potential developmental and metabolic risks associated with prenatal caffeine exposure and further highlight the translational relevance of the current zebrafish model. Although zebrafish provide an established vertebrate model for developmental toxicology, direct waterborne exposure does not replicate the pharmacokinetics of human oral caffeine and glucose consumption. Consequently, the present findings should be interpreted within the context of the zebrafish embryo model rather than as direct evidence of human fetal responses.

Recent zebrafish studies have demonstrated that caffeine exposure can alter behavioral responses, locomotor activity, biochemical parameters, sensory development, sleep regulation, respiratory physiology, social behavior, and skeletal development [24,36,37,38,39,40,41,42]. In addition, developmental stage-specific caffeine uptake, metabolism, and xenobiotic processing may influence embryonic susceptibility to caffeine exposure [43,44]. Together with the transcriptomic findings presented here, these observations suggest that caffeine exposure is associated with alterations in multiple developmental, metabolic, neurological, cardiovascular, and physiological pathways during embryogenesis, highlighting the importance of understanding combined dietary exposures such as caffeine and glucose during development.

Although the transcriptomic analyses identified multiple biological pathways associated with caffeine exposure and caffeine plus glucose co-treatment, these findings represent molecular associations rather than direct mechanistic evidence. Functional studies, including gene-specific manipulation and pathway validation, will be necessary to determine whether the observed transcriptomic changes directly contribute to developmental abnormalities such as delayed hatching, reduced heart rate, and pericardial edema.

Several limitations should be considered in the present study. First, a glucose-only treatment group was not included because the developmental and transcriptomic effects of glucose alone have been reported previously by our laboratory [27]. Consequently, the present study cannot definitively distinguish glucose-specific effects from caffeine–glucose interaction effects within the same experimental framework, and the observed differences between the caffeine and caffeine plus glucose groups should therefore be interpreted cautiously. Second, only a single concentration of caffeine and glucose was evaluated. Because a dose-response analysis was beyond the scope of this study, concentration-dependent developmental and transcriptomic effects could not be determined. Furthermore, zebrafish embryos were exposed directly through the aquatic environment, which differs from human oral caffeine and glucose consumption involving gastrointestinal absorption, metabolism, placental transfer, and fetal distribution. Therefore, caution should be exercised when extrapolating these findings to human pregnancy. Third, transcriptomic analysis was performed at a single developmental time point and all embryos were obtained from a single spawning event, which may limit the characterization of dynamic molecular changes during embryogenesis and the generalizability of the findings. Future studies incorporating glucose-only controls, multiple exposure concentrations, temporal transcriptomic analyses, oxidative stress pathways, behavioral assessments, protein-level validation, and multiple independent clutches will provide a more comprehensive understanding of the developmental and molecular effects of combined caffeine and glucose exposure.

## 5. Conclusions

In conclusion, caffeine exposure induced significant developmental, physiological, and transcriptomic alterations during zebrafish embryogenesis, including morphological abnormalities, altered hatch rate, altered heart rate, and widespread changes in gene expression. Transcriptomic analyses revealed differential expression of genes associated with pathways involved in neuronal signaling, embryonic development, metabolism, mitochondrial function, and alternative splicing regulation. Compared with caffeine exposure alone, caffeine plus glucose co-treatment was associated with distinct transcriptomic and physiological responses, suggesting that glucose may influence molecular responses during caffeine exposure. However, because a glucose-only treatment was not included in this study, these findings should not be interpreted as definitive evidence of caffeine–glucose interaction effects. Overall, this study provides new insight into the developmental, physiological, and transcriptomic responses associated with caffeine exposure in zebrafish embryos and identifies molecular differences associated with glucose co-treatment that warrant further investigation through functional studies.

## Figures and Tables

**Table 1:** Functional Transcriptomic Alterations and Biological Interpretation of Representative Differentially Expressed Genes among Caffeine and Glucose Treatment Groups.

Functional Category	Comparison	Representative Genes (log2FC)	Biological Interpretation
**Neuronal Signaling/Neurodevelopment**	Caffeine vs. Control	*cnih2* (+5.28), *nr4a3* (+3.68)	Caffeine altered neuronal signaling and synaptic activity pathways during embryogenesis.
	Caffeine + Glucose vs. Caffeine	*hint1* (+6.44), *pvalb9* (−3.89)	Glucose co-treatment modified neuronal signaling and calcium-associated pathways affected by caffeine.
	Caffeine + Glucose vs. Control	*cnih2* (+6.10)	Persistent neuronal signaling alterations remained in the co-treatment group relative to controls.
**Development/Embryogenesis/Hatching**	Caffeine vs. Control	*he1b* (−8.34), *he1a* (−6.38), *igf1ra* (+5.16)	Caffeine strongly suppressed hatching-associated genes and altered developmental growth signaling.
	Caffeine + Glucose vs. Caffeine	*igf1ra* (−4.15), *emd* (+4.37)	Glucose co-treatment modified growth regulation and embryonic structural development pathways.
	Caffeine + Glucose vs. Control	*he1b* (−8.21), *he1a* (−5.31)	Developmental and hatching-associated pathways remained significantly altered following co-treatment exposure.
**Metabolism/Energy Regulation**	Caffeine vs. Control	*lepb* (+6.40), *rbp2b* (−6.21)	Caffeine exposure disrupted metabolic regulation and retinol-associated developmental metabolism.
	Caffeine + Glucose vs. Caffeine	*slc25a28* (−4.34), *rbp2b* (+4.43), *egln3* (+3.85)	Glucose supplementation reshaped mitochondrial metabolism, oxygen sensing, and vitamin transport pathways.
	Caffeine + Glucose vs. Control	*lepb* (+6.07)	Metabolic signaling pathways remained elevated in the co-treatment condition.
**Immune/Inflammatory Signaling**	Caffeine vs. Control	*irf4b* (+6.34), *il12rb2* (+5.40)	Caffeine activated immune-associated transcriptional and cytokine signaling pathways.
	Caffeine + Glucose vs. Caffeine	*saa* (−3.89)	Glucose co-treatment partially altered inflammatory signaling responses induced by caffeine exposure.
	Caffeine + Glucose vs. Control	*irf4b* (+6.97)	Immune regulatory pathways remained strongly enriched in the co-treatment group.
**Protein Turnover/Stress Response**	Caffeine vs. Control	*psme4a* (+5.28), *ctslb* (−6.86)	Caffeine altered proteasome regulation and lysosomal protein degradation pathways.
	Caffeine + Glucose vs. Caffeine	*psme4b* (−4.50), *rnf25* (−4.42), *fkbp1ab* (−3.99)	Glucose co-treatment influenced protein degradation, folding, and stress-response pathways.
	Caffeine + Glucose vs. Control	*rpl39* (+5.48)	Enhanced translational and ribosomal activity was observed following co-treatment exposure.
**DNA Repair/Chromatin/Cell Cycle Regulation**	Caffeine vs. Control	—	Limited enrichment of major DNA repair genes among top-ranked DEGs.
	Caffeine + Glucose vs. Caffeine	*nasp* (+7.16), *abraxas2a* (+4.25), *naa10* (+4.70), *dohh* (+4.79)	Glucose co-treatment strongly influenced chromatin remodeling, DNA replication, and genome stability pathways.
	Caffeine + Glucose vs. Control	*taf8* (+6.89), *dmtf1* (+5.45)	Co-treatment altered transcriptional regulation and cell cycle-associated signaling pathways during embryogenesis.

## Data Availability

The RNA sequencing datasets generated during this study have been deposited in the NCBI Gene Expression Omnibus (GEO) under accession number GSE334680. The GEO repository contains the raw FASTQ files, processed gene expression count matrices, sample metadata, differentially expressed gene (DEG) tables, Gene Ontology (GO) enrichment results, Kyoto Encyclopedia of Genes and Genomes (KEGG) pathway enrichment results, and alternative splicing (AS) event tables.

## Abbreviations

The following abbreviations are used in this manuscript:

GDM	Gestational Diabetes Mellitus
MAPK	Mitogen-Activated Protein Kinase
hpf	post-fertilization
GO	Gene Ontology
KEGG	Kyoto Encyclopedia of Genes and Genomes
SE	Skipped Exons
A5SS	Alternative 5′ Splice Sites
A3SS	Alternative 3′ Splice Sites
MXE	Mutually Exclusive Exons
RI	Retained Introns
DEGs	Differentially Expressed Genes
